# Analysis of Roll Pass Wear in the Railway Rail Rolling Process

**DOI:** 10.3390/ma18225131

**Published:** 2025-11-11

**Authors:** Piotr Szota, Sebastian Mróz, Andrzej Stefanik, Tomasz Zygmunt, Marcin Bołda

**Affiliations:** 1 Faculty of Production Engineering and Materials Technology, Czestochowa University of Technology, 42-201 Częstochowa, Poland; sebastian.mroz@pcz.pl (S.M.); andrzej.stefanik@pcz.pl (A.S.); 2 Arcelor Mittal Poland S.A. Zakład Huta Królewska, ul. Metalowców 13, 41-500 Chorzów, Poland; tomasz.zygmunt@arcelormittal.com (T.Z.); marcin.bolda@arcelormittal.com (M.B.)

**Keywords:** hot rolling process, roll pass design, wear, rail, FEM, numerical modelling

## Abstract

The rolling process is one of the most efficient methods for manufacturing long products with both regular and more complex cross-sectional shapes, the latter requiring the development of geometrically complex roll passes. Railway rails are one such product, manufactured at ArcelorMittal Poland S.A., Huta Królewska plant. During the rolling process, the roll passes are subject to wear due to several concurrent phenomena, such as mechanical fatigue, abrasive wear, and thermal fatigue. The determination of roll wear can be based on the experience of personnel and statistical data from previous production runs. It is also possible to determine roll wear through numerical modelling using Archard’s wear model. The aim of this paper is to define a methodology for the quantitative and qualitative determination of roll wear, as well as to establish a wear coefficient dependent on the type of plastic forming process. This will enable the development of a new roll pass design for railway rails that takes into account the durability of the roll passes.

## 1. Introduction

The European Union is intensively striving to mitigate the effects of climate change, which are largely caused by emissions-intensive transport. This sector accounts for over 20% of global carbon dioxide emissions, with road transport, including passenger services, constituting as much as three-quarters of this total. In terms of cost, rail freight transport over longer distances is typically more competitive than road transport. The advantage of rail transport is growing within the EU, making the development of both passenger and freight rail a priority [1].

A key element in the modernization of rail transport in EU countries is the expansion and upgrading of track networks, aimed at increasing the capacity and speed of freight trains, which in Central and Eastern Europe average only 20–30 km/h. The introduction of unified standards for the shape and dimensions of railway rails, European Standard EN 13674-1 [2], is intended to improve the quality of tracks and eliminate technical problems related to the operation of trains within the EU’s transport corridors [1,3,4].

The requirements set by European standards mean that 50E2 rails, among others, must be manufactured to a narrow dimensional tolerance. Therefore, developing the roll pass design requires the use of advanced computer tools for theoretical analysis based on the finite element method, which allows for, inter alia, the determination of the plastic flow of metal during rolling and the wear of the roll passes [5,6].

**Figure 1:**
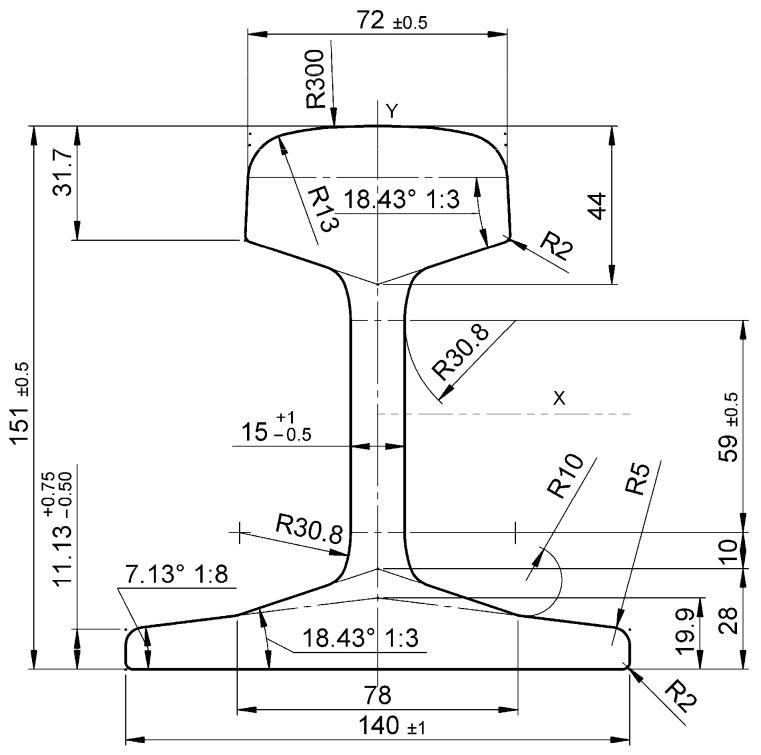
Dimensions of 50E2 rail cross-section [2].

One of the many rails used for track construction in the EU is the 50E2 rail, shown in Figure 1 [2].

The rail shown in Figure 1 is characterised by having the widest foot of all rails, with a mass of up to 50 kg/m. It also has a narrower web and relatively small fillet radii on the foot and head. Among the most important dimensions specified in standard PN EN 13674-1:2011+A1:2017 [2] within the restricted tolerance class (class X) are the rail height and head width, for which the dimensional deviation cannot exceed ±0.5 mm.

Railway rails are primarily produced in large section rolling mills, which is dictated by the significant overall dimensions of the passes. The literature [7,8,9] on rail rolling describes various rolling schemes, which occur in a specific sequence; however, the rolling scheme itself must be adapted to the conditions of the individual rolling mill, particularly concerning the availability of auxiliary equipment. The processes for rolling rails typically use between 6 and 10 passes, depending on the size and type of the rail.

The intensification of rail rolling processes currently focuses on both introducing new product ranges to meet market demands and modifying the roll pass designs used in the manufacturing process to reduce roll wear. This is aimed at obtaining larger quantities of finished products that meet strict acceptance standards while reducing the need for the reconditioning of work rolls [10,11]. In the hot rolling process, work rolls must be resistant to cracking and, to a certain extent, to sudden kinetic changes (impacts from the rolled stock), but above all to the continuous process of surface degradation resulting from friction and abrasion. Furthermore, the wear of the passes is increased by the use of deep incisions (cutters) designed for the initial division of the rolled material volume (in the case of rails, the division into the head and foot) [12,13]. As early as the 1960s, numerous studies on the non-uniformity of wear in roll passes were published; research was conducted on the wear of rolls used for rolling channel sections, which determined that for the process of rolling in passes, the maximum wear on the circumference of the pass must be taken into account [13,14]. This allows for the determination of the depth to which the rolls must be reconditioned after a rolling campaign is completed [14,15].

In the analysis of the roll wear process, Spuzic [13] identifies a number of key factors and mechanisms: abrasion, thermal fatigue, corrosion (fatigue, stress), and wear resulting from the adhesion of rolled material particles to the rolls. Abrasion is one of the dominant factors influencing the roll wear process [16,17]. Abrasion can be understood as material loss between two bodies (the work roll and the hot-rolled material) or three bodies (including the scale forming on the surface of the rolled stock). Given the common presence of hard, low-plasticity oxide scales on hot steel surfaces, three-body wear can be expected to be the most prevalent mechanism of roll wear [18,19]. Another widely known mechanism of roll wear is thermal fatigue of the roll [16,18,20,21]. Each point on the roll surface is alternately heated by contact with the hot material and then cooled with water. As a result, compressive and tensile stresses are generated at the frequency of the roll’s rotation. If the compressive stress exceeds the yield strength of the roll material during the heating stage, the outer layer will deform plastically. Subsequently, during cooling, a high tensile stress is applied to the roll surface without plastic deformation, as the ductility of the roll material is insufficient at these lower temperatures. Various forms of corrosion, e.g., fatigue corrosion or stress corrosion, are also responsible to some extent for roll wear [17]. However, as described in the work by Ohnuki [22], oxides formed by high-temperature oxidation on the roll surfaces can improve wear conditions: at specific temperatures, dependent on the roll material grades, a hard and smooth, black magnetite scale layer forms on the roll surface, and excellent resistance to abrasive wear is achieved.

In [23], the authors also analysed the wear mechanisms in the rail grinding process and the effect of speed on the formation of the white layer. The study demonstrated a significant effect of speed on the wear mechanisms, and observed increases in residual stresses and the number of local cracks.

In recent years, several research papers have presented concepts for predicting the wear of both smooth [24,25,26] and grooved [27,28] rolls, which utilise various wear mechanisms correlated with numerical studies and actual results obtained in industrial trials. In works [29,30], it was proposed that Archard’s wear model [31,32] could be applied to assess roll wear in groove rolling processes. This model is used in the Forge NxT 2.1 (Transvalor, Biot, France) software for the numerical modelling of plastic forming processes [33] and was employed to evaluate the wear and optimise the shape of slitting passes used in the three-strand rolling process of 16 mm diameter ribbed bars. The authors, using actual wear results from work rolls, determined the wear profile of the pass with a 3D scanner, which was then related to the specific work of friction forces calculated in numerical studies. The correlation of experimental and theoretical results made it possible to determine coefficients for predicting roll wear according to the Archard’s model. Within the conducted research, roll wear during rolling was analysed using numerical modelling, and the results obtained were verified in experimental rolling trials. During the rolling of railway rails, the initial shaping and dividing passes for the foot and head are subjected to significant loads. A series of phenomena occur on the surface of the rolls, ranging from rapid heating and cooling of the surface layers to the occurrence of high friction forces with a significant difference between the peripheral speed of the roll and the speed of the moving stock. During rolling, despite considerable differences in peripheral speed between the roll surface and the moving stock in the forward and backward slip zones, there is also a sticking zone (adhesion zone) where the metal contacts the rolls without slip. In this zone, microstructural phase transformations occur on the roll surface due to high pressure and temperature, causing austenitisation of the surface. These conditions are conducive to the bonding of the roll surface with the rolled material, and the subsequent loss of temperature causes grains of the roll material to be torn out, leading to irregular material loss. The intensity of this process depends on many factors, such as the roll material, the rolled material, and the parameters of the rolling process. The simultaneous occurrence of all these factors makes it extremely difficult to determine the influence of each one individually.

## 2. Research Methods

As part of the research conducted, a roll pass design was developed for rolling the 50E2 rail, tailored to the operating conditions of the ArcelorMittal Poland S.A., Huta Królewska plant. The rolling mill operates in an in-line arrangement and has two three-high stands with a roll pitch diameter of 815 mm and one two-high stand with a roll diameter of 780 mm. In stands 1 and 2, reversing rolling is performed, alternately in the upper pair of rolls and then in the lower. In stand 3, single-direction rolling is carried out as a finishing pass. Furthermore, the rolling mill is equipped with auxiliary equipment in the form of manipulators and a tilting device before stand 1. This mill configuration allows for the rails to be rolled in 8 to 10 passes. In the first stage of the work, for the rolling mill characteristics defined above, a roll pass design was developed using engineering methods. The geometry of the passes and the three-dimensional models of the rolls were prepared in CAD software (Rhinoceros v4 SR9). In the next stage of the work, the correctness of the shape of the individual passes was verified using FEM simulation, and appropriate corrections were made to the pass shapes in order to obtain a finished product with the assumed dimensional accuracy.

The developed roll pass design for the 50E2 rail consists of a sequence of 10 passes. The first 4 passes are used to shape the rectangular stock into an inverted “hat” shape; pass 5 performs the important function of dividing the material volume into the portions intended for the rail head and foot. The initially divided stock is then shaped in passes 6 and 8, the task of which is to form the head and foot, as well as to gradually shape the rail web by reducing its thickness and increasing the distance between the head and the foot. At this stage of rolling, the thickness of the foot flanges is also reduced along with their vertical reduction. Passes 6 and 7 are coupled, which means that the upper groove of pass 6 is the same as the lower groove of pass 7. This makes it possible to limit the space allocated for these passes (the working area of the rolls), though a consequence is the attainment of, for example, smaller elongation coefficients. In passes 9 and 10, the final shape of the rail (head and foot) is imparted, which is characterised by the appropriate inclinations and radii, a feature that is also important during the straightening of the rail in roller straighteners.

**Figure 2:**
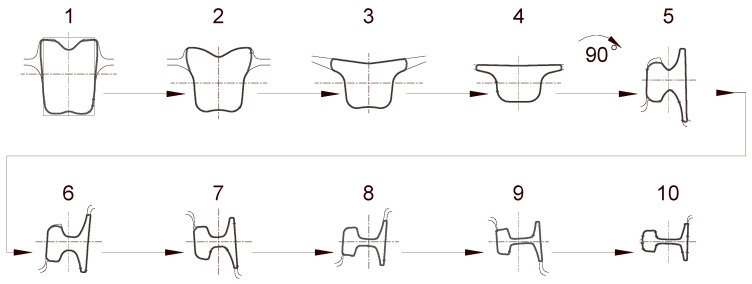
Roll pass design (10 grooves) to the rolling process of the 50E2 rail.

**Figure 3:**
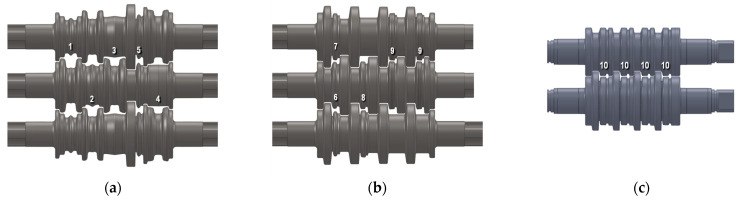
Location of the 50E2 rail rolling grooves: (**a**) stand 1, (**b**) stand 2, (**c**) stand 3.

The schematic of the developed roll pass design is shown in Figure 2. The assembly of the rolls with the arranged passes is shown in Figure 3.

The feedstock for rolling the 50E2 rail was a 165 mm × 265 mm billet produced in a breakdown mill, which was heated in a furnace to a temperature of 1180 °C before rolling. Then, after being rolled in the scale breaker, the billets are introduced into the first stand of the in-line arrangement and rolled at a speed of 3 m/s.

During the development and production planning, it is important to determine the service life of the rolls and the effectiveness of the pass design (optimisation in terms of their service life). Engineering calculations do not allow for the determination of local rolling parameters and the loading on the passes; this is made all the more difficult due to the different metal flow velocities in the section passes. The use of numerical modelling with the finite element method significantly helps in solving this problem. The precise determination of roll wear in numerical modelling requires the most accurate possible input of initial data for the computer simulation. This paper presents the data most relevant to determining roll wear.

As part of the work carried out, a verification of the developed roll pass design was performed using numerical modelling.

### 2.1. Computer Simulations

Computer simulations of the steel samples bending were carried out using an visco-plastic model of material deformation, in which the mechanical state of the deformed material is described using the Norton–Hoff law (3) [34,35]:

(1) $$S_{ij}=2K(T,\dot{\bar{\varepsilon }},\bar{\varepsilon }){\left(\sqrt{3}\dot{\bar{\varepsilon }}\right)}^{m-1}{\dot{\varepsilon }}_{ij}$$

where $S_{ij}$—stress tensor deviator, $\dot{\bar{\varepsilon }}$—strain rate intensity, ${\dot{\varepsilon }}_{ij}$—strain rate tensor, $\bar{\varepsilon }$—strain intensity, *T*—temperature, *K*—stress dependent consistency *σ*, *m*—coefficient describing the hot deformation of the metal (0 < *m* < 1).

For the tests, R260 grade steel was used (Table 1), the parameters of which were adopted on the basis of plastometric tests conducted at the Częstochowa University of Technology using a Gleeble 3800-GTC physical simulator from Dynamic Systems Inc. (Poestenkill, NY, USA). The plastometric compression tests were carried out in a plane strain state using rectangular cuboid samples measuring 20 mm × 10 mm × 10 mm. This state of strain best represents the rolling process.

Based on the plastometric tests performed in the true strain range from 0 to 1.2, for strain rates of 0.1 s^−1^, 1 s^−1^, 10 s^−1^ and 100 s^−1^ at temperatures of 900 °C, 1000 °C, and 1100 °C, flow curves were developed.

**Figure 4:**
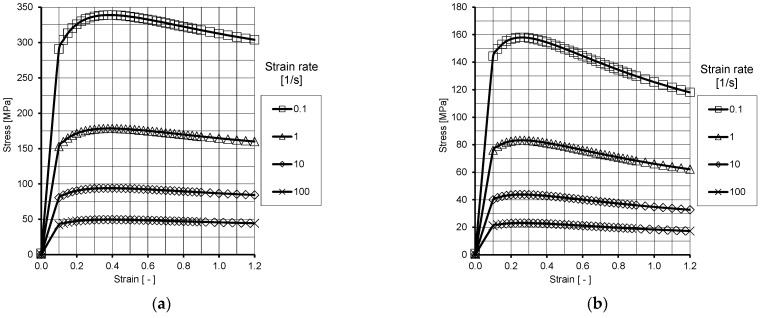
Plastic flow curve of R260 steel grade obtained during Gleeble 3800 test: (**a**) 900 °C, (**b**) 1100 °C.

Due to the fact that the approximation results deviated significantly from the actual plastometric curves, a hybrid method was adopted for inputting the curves. The hybrid method involves inputting the actual data within the range of the plastometric tests performed and data obtained from approximation for the range of very low strain rates (0.0001 s^−1^) and high strain rates (>100 s^−1^) calculated from Equation (2) using the factors from Table 2. Figure 4 graphically shows the behaviour of the yield stress described by Equation (2) [37].

(2) $$\sigma_{f}=A\cdot e^{m_{1}\cdot T}\cdot \varepsilon^{m_{2}}\cdot {\dot{\varepsilon }}^{m_{3}}\cdot e^{\frac{m_{4}}{\varepsilon }}\cdot {\left(1+\varepsilon \right)}^{m_{5}\cdot T}\cdot e^{m_{6}\cdot \varepsilon }\cdot {\dot{\varepsilon }}^{m_{7}\cdot T}\cdot T^{m_{8}}\;[\mathrm{MPa}]$$

where $\sigma_{f}$—flow stress, ε—true strain, $\dot{\varepsilon }$—strain rate, *T*—temperature, *m*_1_–*m*_8_—function coefficients.

The computer simulation also requires the following to be entered: friction coefficient μ—0.4, friction factor m = 0.8; ambient temperature—20 °C; Poisson coefficient—0.3; rolling velocity 3 m/s, specific heat = 480 J/(kg·K), density = 7850 kg/m^3^, conductivity = 29.9 W/(m·K), heat transfer coefficient 3000 W/(m^2^·K). The value of a good set is based on the authors’ long-standing experience, both in industrial research and in available literature [38,39,40]. The presented results of previous studies on rolling processes confirm the validity of the values adopted for this study. A schematic of the geometrical model used in the numerical calculations is shown in Figure 2.

The friction conditions prevailing between the metal and the rolls are described by the Coulomb and Tresca friction models (3) and (4), in which appropriate coefficient values are assumed:

(3) $$\tau_{j}=\mu \cdot \sigma_{n}\;\mathrm{for}\;\mu \cdot \sigma_{n}<\frac{\sigma_{0}}{\sqrt{3}}$$

(4) $$\tau_{j}=m\frac{\sigma_{0}}{\sqrt{3}}\;\mathrm{for}\;\mu \cdot \sigma_{n}>m\frac{\sigma_{0}}{\sqrt{3}}$$

where *τ_j_*—vector of specific friction forces, *σ*_0_—base stress, *σ_n_*—normal stress, *μ*—friction coefficient, *m*—friction factor.

The model described by Equations (3) and (4) accurately describes the contact conditions between the rolls and the rolled stock. The Tresca condition (4) defines the moment of transition from the slip zones in the roll gap to the narrow sticking zone, in which the friction stresses are so high that there is no slip between the roll and the material. This model is perfectly suited to modelling the slip between the roll and the material. This is significant due to the model’s application in estimating roll wear in the rolling process.

To determine the temperature field, the software uses a differential equation describing temperature changes in transient heat conduction. This is a quasi-harmonic equation in the form of:

(5) $$\frac{\partial }{\partial x}\left(k_{x}\frac{\partial T_{s}}{\partial x}\right)+\frac{\partial }{\partial y}\left(k_{y}\frac{\partial T_{s}}{\partial y}\right)+\frac{\partial }{\partial z}\left(k_{z}\frac{\partial T_{s}}{\partial z}\right)+\left(Q-c_{p}\rho \frac{\partial T_{s}}{\partial t}\right)=0$$

In the equation, *k_x_*, *k_y_* and *k_z_* are functions of the distribution of anisotropic thermal conductivity coefficients in the *x*, *y*, *z*, *T_s_* is a function that represents the temperature distribution in the zone under consideration, *Q* represents the function of the distribution of the heat generation rate due to deformation, *c_p_* is a function of the distribution of the specific heat of the metal, and *ρ* is a function of the distribution of its density. The boundary conditions adopted were combined boundary conditions of the second and third kind, which can be written in the form:

(6) $$k_{x}\frac{\partial T_{s}}{\partial x}l_{x}+k_{y}\frac{\partial T_{s}}{\partial y}l_{y}+k_{z}\frac{\partial T_{s}}{\partial z}l_{z}+q+\alpha_{k}T_{s}=0$$

In the equation, *l_x_*, *l_y_*, *l_z_* are the directional cosines of the normal to the stock surface, *q* represents the heat flux on the surface of the cooled zone, and *α_k_* represents the convective losses. Equation (5) and the boundary condition (6) uniquely define the problem of temperature distribution in the deformed material.

The computer models of the rolls were constructed from a surface mesh of triangular elements, whereas the feedstock consisted of tetrahedral elements. The number of elements was in the range of 60,000 to 100,000. Remeshing of the finite element mesh occurred automatically in the event of excessive deformation of a tetrahedral element.

Obtaining realistic results for the distributions of rolling parameters, such as the state of stress, requires the determination of the temperature field. For this reason, the rolling process was modelled starting from the exit of the stock from the heating furnace and through the rolling in individual stands, taking into account the duration of the intervals between passes associated with the transport of the rolled stock. This time increased in accordance with the length of the stock after successive passes. This allowed for the non-uniformities of temperature and their influence on the state of stress and strain to be taken into account. The state of strain was not carried over to the next pass due to the occurrence of dynamic recrystallisation.

The specified rolling conditions and the parameters obtained allowed for the determination of the specific work of friction forces, which is a component of the Archard’s model that defines wear. The general form of the wear model is defined by the equation [30]:

(7) $$wear=k_{w}\int_{0}^{t}\frac{\sigma_{n}v_{s}}{H(T)}dt\;[\mathrm{mm}]$$

where *k_w_*—wear coefficient, *v_s_*—tangential slip velocity of the metal on the tool surface, *t*—time, *H*(*T*)—hardness of the tool at a specific temperature.

In this model, it was assumed that under abrasive wear conditions, the volume of material *V* removed from a unit area of the tool is directly proportional to the normal stress *σ_n_* acting on the tool surface and the slip velocity vs, and inversely proportional to the hardness *H* of the material subject to wear (in this case, the temperature-dependent hardness of the tool).

Based on numerical calculations, it is possible to determine the unit work of friction forces (UWFF) and subsequently the quantitative roll wear.

(8) $$UWFF=\int_{0}^{t}\sigma_{n}\cdot v_{s}dt\;[\mathrm{mm}\cdot \mathrm{MPa}]$$

Since the friction stress *τ_j_* is the product of the normal stress *σ_n_* and the friction coefficient *μ*, as written in Equation (8), the integral has a physical meaning and is equal to the specific work of friction forces per unit area of contact between the metal and the tool.

A qualitative assessment of the wear of the passes can be made based on the distribution of the value of the specific work of friction forces on the roll surface, whereas determining the quantitative roll wear requires knowledge of the roll wear coefficient, defined as *k_w_* in Equation (7). This coefficient encompasses a series of physical phenomena [13,30,41] that take place during the contact of the hot metal with the rolls; however, describing them with mathematical models is extremely difficult due to the occurrence of different rolling conditions. The literature reports values of the wear coefficient kw in the range 10^−7^ to 10^−3^ [41,42,43]. This range is wide, which prevents a quantitative assessment and necessitates the determination of this coefficient.

### 2.2. Experimental Testing Methodology

**Figure 5:**
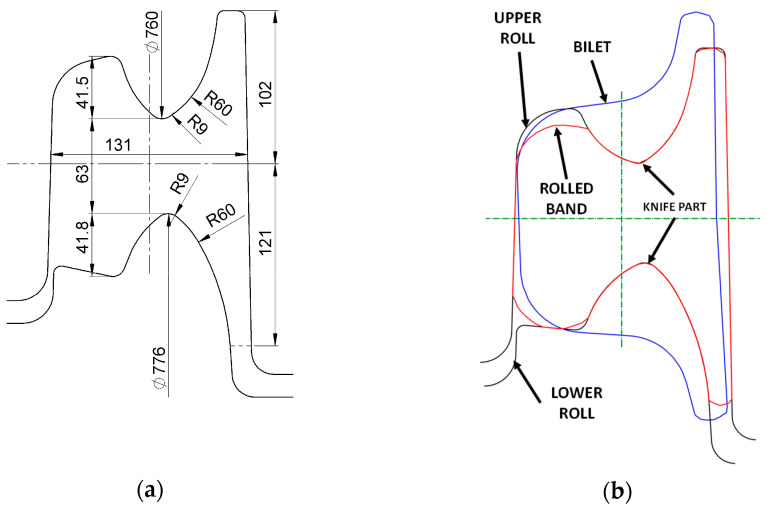
Roll pass 5: (**a**) shape and dimension, (**b**) filling of rolling groove.

Experimental investigations concerning the wear of rolls in the railway rail rolling process were conducted to verify the numerical calculations and to enable the determination of the wear coefficient. As part of the research, pass 5, shown in Figure 2 and Figure 3, was analysed. This pass is the one that performs the initial division of the stock into the rail head and foot, which causes high pressures and high slip velocity values between the rolls and the stock in the cutter region. For this reason, this area of the pass was subjected to detailed analysis. As part of the experiment, 50E2 rail was rolled, for which a batch of 60 pieces of feedstock with a rectangular cross-section of 260 mm × 165 mm and a length of 5 m was prepared. The billet mass was 1683.8 kg, and the total mass of the rolled feedstock was 101 Mg. The pass was machined on rolls made of GS-18CrMo910 cast steel, which allows for the reconditioning of the rolls by hardfacing. The use of such a material is associated with lower wear resistance resulting from its lower hardness of 390 HV compared to cast iron rolls. The shape of pass 5 and the roll diameters are shown in Figure 5.

The diameter of the bottom roll was 776 mm, while that of the top roll was 760 mm. The elongation coefficient of the stock was 3.14; therefore, the length of the rolled stock in this pass was 15.7 m. The total length of the rails from the 60 billets was 1777 m. Based on the knowledge of the roll diameters and the length of the stock in pass 5, the number of roll rotations was calculated (Table 3).

Based on the information gathered from the computer simulations and the experimental rolling, it was possible to determine the roll wear coefficient, which allows for the quantitative determination of roll wear.

## 3. Results of the Conducted Tests

**Figure 6:**
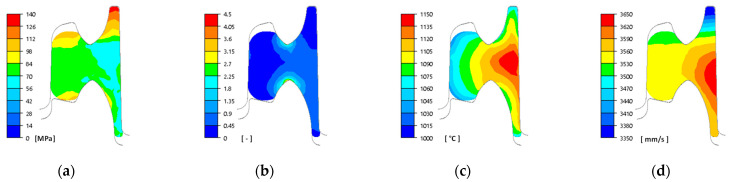
Distribution of rolling parameters: (**a**) effective stress, (**b**) equivalent strain, (**c**) temperature, (**d**) velocity of metal flow.

As part of the research work, numerical modelling of the rolling process in pass 5 was carried out, the aim of which was to reduce the wear of the rolls, particularly in the cutter regions of the pass under investigation. These parts of the pass are exposed to the highest pressures at a high slip velocity resulting from the complex shape of the pass. The cutter region of the pass most often has a small fillet radius of 5 mm, which is prone to chipping (due to the impact of the rolled stock) or abrasive wear during rolling. For this reason, an attempt was made to change the radius to a larger one. A limiting factor in increasing the radius is the increase in pressure on the rolls, and the bite conditions of the stock by the rolls also deteriorate. The numerical modelling was carried out for three variants of the cutter region of the pass: Variant I—5 mm, Variant II—7 mm, and Variant III—9 mm. Based on the numerical modelling, distributions of the main rolling parameters were obtained, example distributions of which for Variant III are shown in Figure 6.

Based on the data presented, a high degree of non-uniformity in the plastic flow during rolling in this pass can be observed (Figure 6). The values of effective stress in the outermost regions of the head and foot are the highest due to different values of strain, temperature, and flow velocity (Figure 6a). High values of effective stress are visible in the freely formed regions of the head and are the result of the difference in flow velocities. The metal in the cutter regions of the pass pulls the material in the outermost regions of the head, which causes high stress values (Figure 6d). The lower temperature in the near-surface regions causes higher stress values (Figure 6c). Due to the greatest reduction occurring between the cutter parts of the pass, the highest values of strain intensity are observed in these locations (Figure 6b).

**Figure 7:**
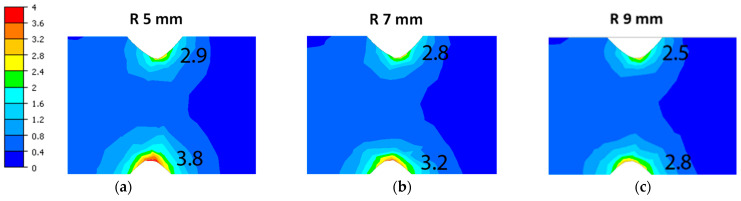
Distribution of equivalent strain during rolling band in 5 pass: (**a**) variant I, (**b**) variant II, (**c**) variant III.

A detailed analysis of the rolling variants using different fillet radii showed the occurrence of lower strain values in the regions of the stock located directly under the influence of this part of the pass groove, as shown in Figure 7.

In the case of a smaller fillet radius at the tip of the bottom and top cutter, a local increase in strain occurs in a smaller area, hence the strain intensity values are higher (Figure 7a) than in the case of using a larger radius for the dividing cutters (Figure 7c).

**Figure 8:**
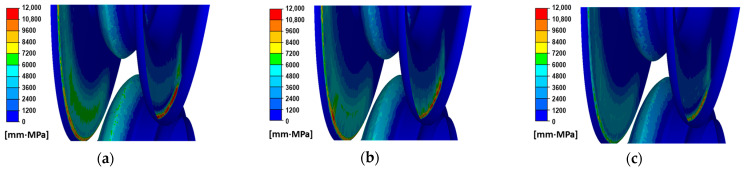
Distribution of unit work of friction force on a roll surface: (**a**) variant I, (**b**) variant II, (**c**) variant III.

The numerical modelling of the rolling process performed allowed for the determination of the specific work of friction forces, which defines the qualitative wear of the rolls. Figure 8 shows the distribution of the specific work of friction forces for the three variants.

**Figure 9:**
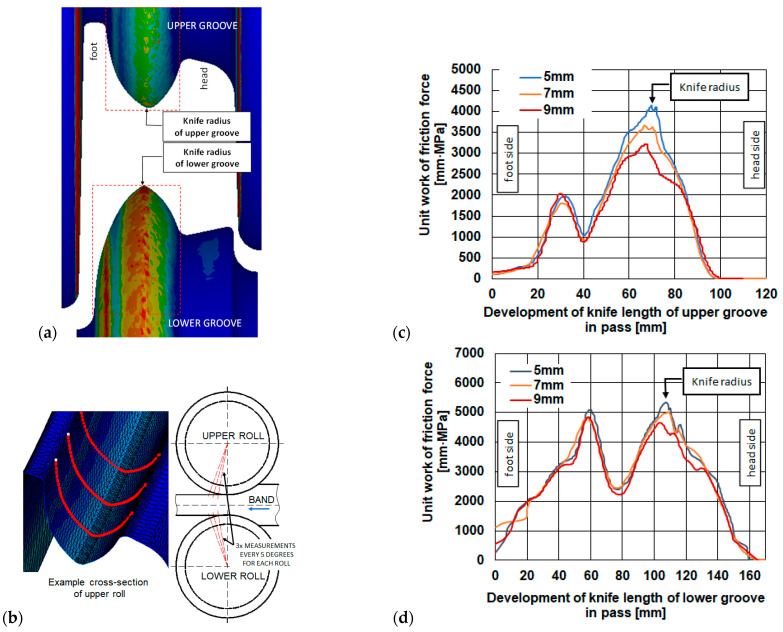
Wear of the separating pass for rolling the rail: (**a**) distribution of unit specific work of friction forces, (**b**) places of making a cross-section, (**c**) unit work of friction forces of the upper groove knife, (**d**) unit work of friction forces of the lower groove knife.

Figure 8 shows the roll wear for the three variants of the dividing cutter radius. In the case of the smallest radius, the highest values of the specific work of friction forces were observed. High values of the specific work of friction forces are observed on the lateral surfaces of the pass. However, such high wear on the passes occurs only during the entry and exit of the stock from the roll gap, and this wear is not significant compared to the wear of the cutter parts in the steady-state rolling process. For the variants shown in Figure 8, a detailed analysis of the distribution of the specific work of friction forces was carried out, as shown in Figure 9a.

For all variants of the cutter radius, for both the bottom and top roll, three cross-sections each were made in the cutter region of the pass, offset from each other by 5° (Figure 9b). The values obtained on the cross-section lines (Figure 9b) were averaged and are shown in Figure 9c (top pass) and Figure 9d (bottom pass).

The calculations performed showed that when using the fillet radius according to Variant I, amounting to 5 mm, the specific work of friction forces is the highest. Increasing the fillet radius causes a reduction in the specific work of friction forces, which means that the wear of the rolls in the areas with lower specific work of friction forces will be smaller. Based on the specific work of friction forces, it can be stated that the use of a 7 mm radius results in 6% less wear, while for a 9 mm radius, the wear was 13% less compared to the cutter having a 5 mm fillet radius. In the remaining areas of the incising region of the cutter, the obtained values of the specific work of friction forces are similar. The geometry of the passes was not changed in these areas.

Determining the quantitative wear of rolls is difficult due to the simultaneous occurrence of many physical phenomena. However, for engineering purposes, roll logistics, and production planning, it is necessary to determine the quantitative wear of the rolls.

Predicting the quantitative wear of rolls requires measurements to be taken of the rolls after an actual rolling campaign.

**Figure 10:**
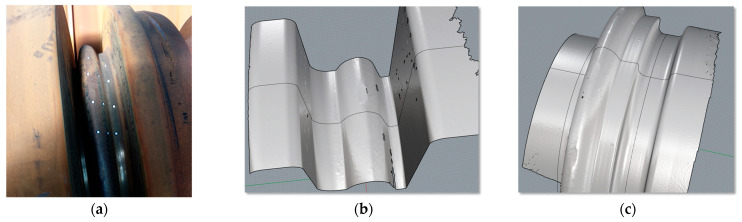
View of the rolls: (**a**) the actual roll after the rolling campaign, (**b**) captured surface fragment of the top roll pass, (**c**) captured surface fragment of the bottom roll pass.

The rolling of the 50E2 rail was carried out using pass 5 according to Variant III with a fillet radius of 9 mm. After rolling 101 Mg of feedstock, the rolls were inspected for wear. The shape of the pass after rolling was captured using a portable EinScan HX laser scanner (Shining3D Hangzhou, China). The technology, which uses a blue laser, allows the scanned surface to be captured with an accuracy of up to 0.03 mm. Figure 10 shows an example of a roll subjected to scanning, as well as fragments of the surface captured using a triangular mesh. The scanned surfaces were constructed from over 500,000 nodes with a spacing of 0.8 mm.

**Figure 11:**
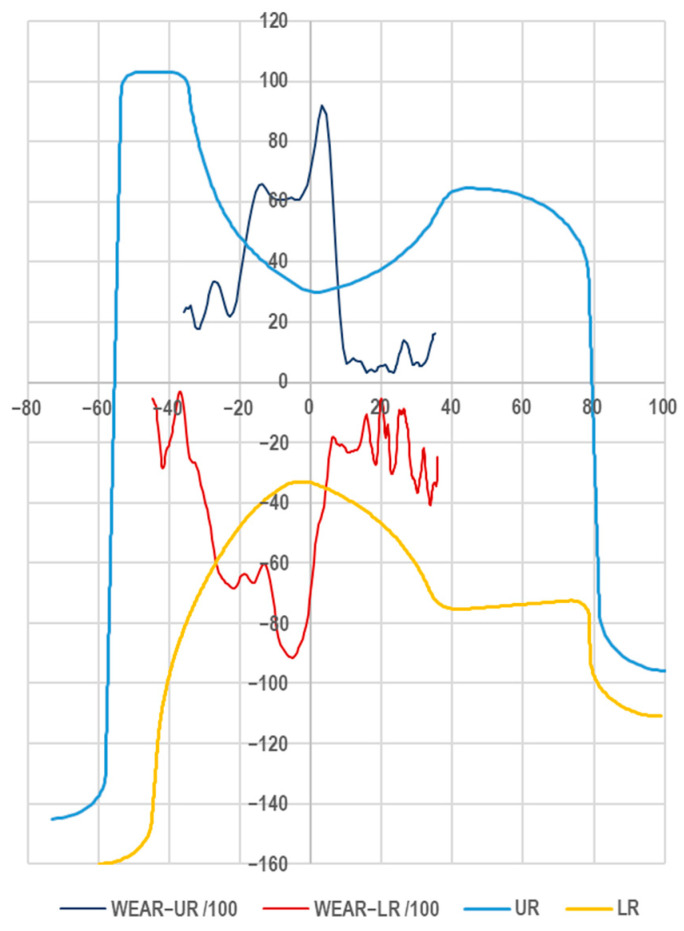
Shape of upper (UR) and lower (LR) roll of pass 5 and knife wear of upper (WEAR-UR) and lower (WEAR-LR) roll.

The roll surfaces captured by scanning were measured in CAD software (Rhinoceros v4 SR9) and then superimposed on the theoretical shape of the pass. The actual shape of the passes is obtained through a machining process of turning, which was carried out on an industrial CNC machine tool (THG 120, Poręba, Poland), which provides a dimensional accuracy of 0.1 mm. The aim of the measurements was to investigate the differences between the theoretical shape of the pass and the worn shape obtained via 3D scanning. Figure 11 shows the theoretical shape as well as the wear, in mm, of the stock-dividing cutter’s shape, this being the difference between the scanned and theoretical surfaces.

During rolling, the lateral surfaces of the passes wear to a significant extent, as evidenced by the distributions of the specific work of friction forces shown in Figure 8. In practice, this wear is the result of the stock being introduced and positioned in the pass immediately before rolling. The wear on the lateral surfaces of the passes is not very reliable due to the non-uniform and unstable surface loading conditions. For this reason, to determine the wear coefficient, only the cutter region of the pass, which is loaded uniformly during rolling, was analysed, as this allows for reliable results to be obtained. Figure 11 shows the pass wear curves (magnified 100×) and the pass geometry, which define the quantitative wear of the dividing cutter across its width. The quantitative wear values for pass 5 were determined based on the averaged values from three geometrical differences between the scanned surface and the theoretical surface. The cross-sections were made in a polar coordinate system relative to the axis of rotation at 5° intervals and were then rotated by the required angle and presented in an XY coordinate system. To capture the pass profile, 500 measurement points were used. The accuracy of the radii capture was 0.01 mm.

**Figure 12:**
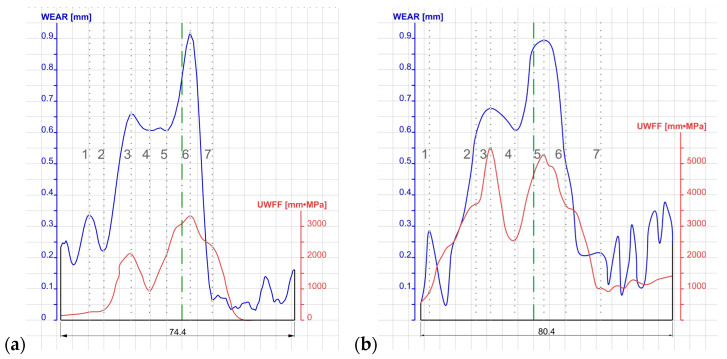
Unit work of friction forces correlated with the actual wear of the knife part of the cut: (**a**) upper roll, (**b**) lower roll.

The data presented in Figure 11 show that reliable wear results for the pass can be observed in the cutter region. For the cutter surfaces, the quantitative roll wear was determined in mm, where the highest values occur at the tips of the cutters, where the loads are greatest. Less quantitative wear was observed in the foot region of the pass, on the arcuate surfaces, due to the fact that during the rolling of the metal in the foot region, the foot is compressed. Because the pass strongly restricts the flow of metal, some of the metal flows transversely towards the head. This causes limited contact or only a slight load on the head surface on the side of the dividing cutters, which translates into low wear values on these surfaces. Linking the specific work of friction forces to the real-world wear through the wear coefficient demanded that these data be precisely superimposed according to the cutter width of the top and bottom passes, as depicted in Figure 12.

The data shown in Figure 12 were scaled due to the large discrepancy in their orders of magnitude. The specific work of friction forces was divided by 100, whereas the roll wear was magnified 100 times. On the basis of the data prepared in this way, cross-section lines were determined, defining the characteristic wear locations of the dividing cutters of the top pass (Figure 12a) and the bottom pass (Figure 12b). Based on the determined values of WEAR and UWFF in the individual cross-sections, taking into account the rolling parameters HV and the number of rotations determined from the length of the roll gap, the wear coefficient *k_w_* was determined. A summary of the data is shown in Table 4 and Table 5 for the top and bottom rolls.

Based on the analysis performed, it was found that for certain cross-sections, the values of the *k_w_* coefficients deviated extremely from the other results. For this reason, the lowest and highest values were not taken into account in the further analysis (they are marked with grey fields). In the case of the top roll pass, large discrepancies occurred in the outermost cross-sections where the pass walls approach a vertical orientation or where the contact between the metal and the rolls was smaller due to the transverse flow of the metal in the pass. In the case of the bottom roll pass, large inconsistencies were obtained in cross-sections 1 and 3, caused by non-uniform forming conditions in the regions of the stock’s foot.

The determination of wear coefficients at different locations was aimed at checking what discrepancies occur across the width of the cutter regions of the pass. Table 4 and Table 5 show the determined roll wear coefficients, which were 4.24·10^−4^ for the top roll and 2.03·10^−4^ for the bottom roll, respectively. The average value determined on the basis of the research was 3.13·10^−4^.

**Figure 13:**
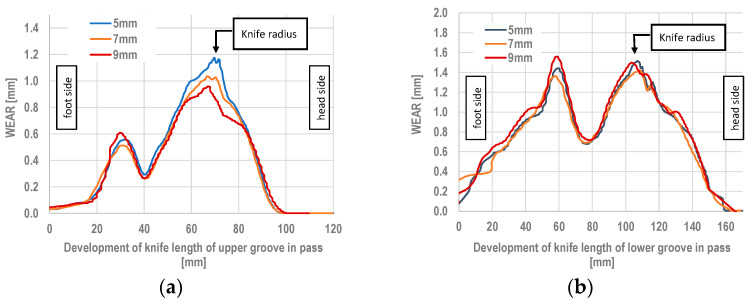
Quantitative wear of the knife part of the upper roll (**a**) and the lower roll (**b**).

By adopting the average value of the wear coefficient determined from the numerical modelling and experimental rolling, the quantitative wear of the rolls can be determined for the other variants of the pass geometries. The results of the calculations are shown in Figure 13.

Based on the research conducted, it was possible to determine the quantitative wear of the rolls, as shown in Figure 13. The use of numerical modelling allows for the quantitative wear of rolls to be assessed with an average accuracy of 20%.

## 4. Discussion

The results obtained are characterised by significant uncertainty, expressed by the relative error, which means that the wear coefficient in the Archard’s equation encompasses all the phenomena that occur during the contact of the rolls with the rolled stock. It can therefore be concluded that the dependence of wear on the specific work of friction forces is not a linear relationship that would allow the quantitative wear of the rolls to be determined directly. For engineering purposes, knowledge of the wear coefficient is extremely important because it allows for the estimation of the durability of rolls, production planning, and prediction of the workload of the roll lathe shop. These aspects translate into the economics of product manufacturing.

The research results obtained indicate that the precise determination of roll wear, from a research point of view, requires the development of theoretical models of thermal fatigue and the phenomena of adhesion of the rolls to the rolled material, taking into account the austenitisation of the roll surface to a depth of approximately 50 μm. While the existence of these phenomena is known, the mathematical description of the phenomena and their contribution during forming in the roll gap is extremely difficult to observe under real conditions and equally difficult to replicate and measure under real conditions.

**Figure 14:**
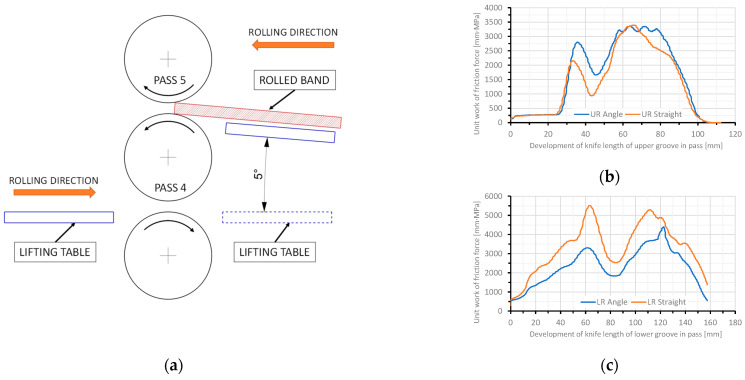
Rolling in a trio rolling mill in two directions using lifting tables: (**a**) rolling scheme, (**b**) unit work of friction forces of the upper roll, (**c**) unit work of friction forces of the lower roll.

As part of the research, an additional analysis of the specific friction work was performed using FEM, which took into account the strand feed angle as in the actual process Changing the feed angle affects the distribution of specific work of friction forces on the upper and lower rolls. Numerical calculations of the rolling process, in which the feed was fed at an angle (Figure 14a), showed that the specific friction work for the upper roll (Figure 14b) is greater than for rolling with the strand fed straight in.

For the lower roll (Figure 14c), the specific friction work when the strand is fed into the rolls at a 5° angle is lower. Assuming actual wear values and the calculated specific friction work for the strand fed at a 5° angle, the wear coefficient values will The calculated wear coefficients *k_w_*, based on the average unit value of the friction force work, for this case are 3.1·10^−4^ for the upper roll and 2.6·10^−4^ for the lower roll. Determining the wear coefficient more precisely requires analysing a larger number of rolling cases, which will be the subject of subsequent work.

## 5. Conclusions

The numerical investigations carried out using Forge NxT 2.1 (Transvalor, Biot, France) allow for the determination of quantitative wear with an accuracy of 20%.

The average wear coefficient *k_w_* determined on the basis of the research conducted was 3.13·10^−4^. Obtaining more accurate results for roll wear requires additional experimental research to be carried out for a larger rolling campaign.

The influence of the strand feed angle on the unit work of friction forces during rolling in pass 5 was observed. Taking the strand feed angle into account reduces the differences in the calculated wear coefficients for the upper and lower rolls.

Based on the research conducted, it was found that the wear coefficient does not change in a linear manner and should take into account phenomena related to thermal fatigue and the adhesion of metal to the roll at high temperatures.

## Figures and Tables

**Table 1:** Chemical composition steel grade R260 (EN 13674-4 Standard [36]), [%].

Steel Grade	Number	C	Si	Mn	P	S	Cr	V
R260	1.0623	0.75	0.3	0.68	<0.02	0.01	<0.15	<0.03

**Table 2:** Hansel-Spittel function coefficients for R260 steel.

*A*	*m*_1_	*m*_2_	*m*_3_	*m*_4_	*m*_5_	*m*_6_	*m*_7_	*m*_8_
1085.62	−0.0036579	0.313984	−0.278478	−0.0003009	−0.001767	0.33043	0.0004347	0.320209

**Table 3:** Rolling parameters in pass no. 5.

No	Roll	Diameter	Roll Circuit	Number of Revolutions	Length of Rolling Gap
		[mm]	[mm]	[-]	[mm]
1	upper	760	2.3876	355.0831	80.37
2	lower	776	2.4379	347.7618	81.21

**Table 4:** Roller wear coefficient *k_w_* in different cross-sections of the upper roll 5.

Line of Section	Position	UWFF	WEAR	HV	Number of Rotations	*k_w_*	*k_w_. ave.*	δ
	[mm]	[mm·MPa]	[mm]		[rot.]	[-]	[-]	[%]
1	−29.58	259.4	0.3	390	351.4	1.44·10^−3^	4.24·10^−4^	x
2	−25.00	328.0	0.2			**7.45·10^−4^**		−75.8%
3	−16.29	2120.7	0.7			**3.44·10^−4^**		18.8%
4	−10.47	936.5	0.6			**7.20·10^−4^**		−69.9%
5	−4.89	2111.6	0.6			**3.19·10^−4^**		24.7%
6	2.62	3326.9	0.9			**3.06·10^−4^**		27.8%
7	9.63	2358.3	0.1			2.91·10^−5^		x

**Table 5:** Roller wear coefficient *k_w_* in different cross-sections of the lower roll 5.

Line of Section	Position	UWFF	WEAR	HV	Number of Rotations	*k_w_*	*k_w_. ave*.	δ
	[mm]	[mm·MPa]	[mm]		[rot.]	[-]	[-]	[%]
1	−33.40	888.8	0.3	390	351.40	3.54·10^−4^	2.03·10^−4^	x
2	−18.46	3711.4	0.6			**1.77·10^−4^**		12.8%
3	−13.83	5493.4	0.7			1.37·10^−4^		x
4	−6.06	2547.9	0.6			**2.65·10^−4^**		−30.1%
5	3.30	5275.1	0.9			**1.88·10^−4^**		7.5%
6	10.34	3656.0	0.5			**1.51·10^−4^**		25.5%
7	21.44	1009.9	0.2			**2.35·10^−4^**		−15.8%

## Data Availability

The original contributions presented in this study are included in the article. Further inquiries can be directed to the corresponding author.
